# A Novel Cysteine Knot Protein for Enhancing Sperm Motility That Might Facilitate the Evolution of Internal Fertilization in Amphibians

**DOI:** 10.1371/journal.pone.0160445

**Published:** 2016-08-31

**Authors:** Misato Yokoe, Eriko Takayama-Watanabe, Yoko Saito, Megumi Kutsuzawa, Kosuke Fujita, Haruki Ochi, Yuni Nakauchi, Akihiko Watanabe

**Affiliations:** 1 Department of Biology, Faculty of Science, Yamagata University, Kojirakawa, Yamagata, Japan; 2 Institute of Arts and Sciences, Yamagata University, Kojirakawa, Yamagata, Japan; 3 School of Medicine, Yamagata University, Iida-Nishi, Yamagata, Japan; University of Colorado Boulder, UNITED STATES

## Abstract

Internal fertilization ensures successful reproduction of tetrapod vertebrates on land, although how this mode of reproduction evolved is unknown. Here, we identified a novel gene encoding sperm motility-initiating substance (SMIS), a key protein for the internal fertilization of the urodele *Cynops pyrrhogaster* by Edman degradation of an isolated protein and subsequent reverse transcription polymerase chain reaction. The *SMIS* gene encoded a 150 amino-acid sequence including the cysteine knot (CK) motif. No gene with substantial similarity to the *SMIS* was in the data bank of any model organisms. An active site of the SMIS was in the C-terminal region of the 2nd loop of CK motif. A synthetic peptide including the active site sequence bound to the midpiece and initiated/enhanced the circular motion of *C*. *pyrrhogaster* sperm, which allows penetration of the egg jelly specialized for the internal fertilization of this species. The synthetic peptide bound to whole sperm of *Rhacophorus arboreus* and enhanced the rotary motion, which is adapted to propel the sperm through egg coat matrix specialized for arboreal reproduction, while it bound to the tip of head and tail of *Bufo japonicus* sperm, and enhanced the vibratory motion, which is suited to sperm penetration through the egg jelly specialized for the reproduction of that species in freshwater. The polyclonal antibody against the active site of the SMIS specifically bound to egg coat matrix of *R*. *arboreus*. These findings suggest that diversification of amphibian reproductive modes accompanies the specialization of egg coat and the adaptation of sperm motility to penetrate the specialized egg coat, and SMIS acts as the sperm motility enhancer of anurans and urodeles that might facilitate to adaptively optimize sperm motility for allowing the establishment of internal fertilization.

## Introduction

The reproductive modes of amphibians have evolutionarily diversified from external fertilization in freshwater to arboreal fertilization or internal fertilization[1]. Today, internal fertilization occurs in a few anuran species, most urodele species, and all caecilians. It is also common in higher vertebrates, suggesting that the establishment of internal fertilization in amphibians would have greatly contributed to the transition from water to land during vertebrate evolution. However, the mechanism involved in the diversification of reproductive modes and the establishment of internal fertilization is entirely unknown.

Internal fertilization presents a constitutive advantage in allowing the selection of a time and place suited for reproduction, largely as a result of sperm storage by females[2]. In tetrapod females, as sperm are maintained in a quiescent state during the storage period, re-activation of sperm motility is required to achieve internal fertilization. The *in vivo* mechanisms for the re-activation of sperm motility are unknown except in a few species, such as the urodele *Cynops pyrrhogaster*. At the time of fertilization, the motility of *Cynops* sperm stored by females is initiated at the surface of the egg jelly, the outermost egg coat, composed of oviduct-secreted extracellular matrix[3]. The surface of the egg jelly possesses fine structures specialized for the initiation of sperm motility by sperm motility-initiating substance (SMIS)[4]. SMIS induces sperm motility independent of hypoosmolality, the typical trigger for the initiation of sperm motility during external fertilization in amphibians[5], and ensures the success of internal fertilization. SMIS activity is also present in the egg jelly of primitive amphibians that undergo external fertilization, despite the fact that sperm motility is initiated due to the hypoosmolality of freshwater in these species[6,7]. These facts suggest that the role of SMIS was modified, leading it to make an essential contribution to the establishment of internal fertilization in amphibians. In the present study, we identify the *SMIS* gene to address the mechanism of the diversification of the reproductive mode resulting in the establishment of internal fertilization.

## Methods

### Animals

Fifty mature *Cynops pyrrhogaster* were captured by hand in early spring or late autumn in Yamagata prefecture (lat. 38°22’12” N, long. 140°3’57” E), Japan, by permission of landowners and maintained in hibernation at 10°C in the laboratory. Ten mature *Rhacophorus arboreus* were captured by hand in late spring in Yamagata prefecture (lat. 38°4’55” N, long. 140°18’6” E), Japan, by permission of landowners. Some males were provided by Dr. Kubota of Kyoto University. *Bufo japonicus* and *Xenopus laevis* were provided by the Institute of Amphibian Biology, Hiroshima University, or were purchased from PLUSTinc Limited Company, Tokyo, Japan. The anurans were kept at room temperature in the laboratory. Animals were anesthetized in 0.1% MS222 and then pithed. *C*. *pyrrhogaster* sperm were obtained from the vas deferens, while *R*. *arboreus*, *B*. *japonicus*, and *X*. *laevis* sperm were obtained from the testes. To obtain jellied eggs or oviduct-secreted matrix, ovulation was induced in females of *C*. *pyrrhogaster*, *B*. *japonicus and X*. *laevis* by daily injection of 300 IU of human chorionic gonadotropin (Aska Pharmaceutical, Tokyo, Japan). Jellied eggs or egg clutches were obtained from the uterus (*C*. *pyrrhogaster*) or by pressing the stomach (*B*. *japonicus* and *X*. *laevis*). The oviduct-secreted matrix of *R*. *arboreus* was obtained from the ovisac (the most posterior portion of the oviduct) of females captured in their reproductive season (June). The experimental protocol was approved by the committee for animal experiments of Yamagata University (No. 27054), and all animals were treated according to the guidelines for proper conduct in animal experiments in Japan.

### Identification and base sequence analysis of *SMIS* cDNA

An egg jelly extract (JE) was prepared according to a previous study[4]. Isoelectric focusing (pH 3–10) of the JE (above 200 μg) was performed, and a second separation was subsequently carried out in a 10% polyacrylamide gel. The substances present in the JE were electrotransferred to a polyvinylidene difluoride membrane, which was then immunoreacted with an anti-SMIS antibody[4] at 1 μg/ml, followed by immunoreaction with horseradish peroxidase-conjugated anti-mouse IgG (Chemicon International Inc. Billerica, MA) at 1 μg/ml. Specific binding of the antibodies to jelly substances was then revealed using 0.02% diaminobenzidine. A corresponding spot recognized by the anti-SMIS antibody was treated with trypsin, and the N-terminal amino acid sequences of the obtained peptides were analyzed through Edman degradation.

Degenerate primers corresponding to the obtained sequences (PVPYPSYPL and PVSSFDM) were synthesized. Total RNA was purified from the posterior portion of the oviduct of *C*. *pyrrhogaster*, in which ovulation was induced by daily injections of gonadotropin (300 unit). Reverse transcription followed by polymerase chain reaction was performed using the SMARTer^™^ RACE cDNA amplification kit (Takara Bio Inc., Tokyo, Japan). 5’- and 3’- RACE was performed using gene-specific primers and 5’- or 3’- RACE CDS primer A. The base sequences of the amplified DNAs were analyzed with a genetic analyzer (3500; Thermo Fisher Scientific, Waltham, MA). Similarity searches of the cDNA base sequence and the deduced amino acid sequence were performed in the NIH gene or protein data bank and in the data from the *X*. *laevis* J-strain 8.0 genome (Xenbase; http://www.xenbase.org) using the Basic Local Alignment Search Tool.

### *In situ* hybridization

Oviduct of ovulation-induced females by HCG injection (300IU) was dissected and cut into small pieces. They were fixed in 4% paraformaldehyde in PBS. Primers specific for SMIS (Forward: 5’-CACCAATACCCAAGCGAAACAGACGA-3’ and Reverse: 5’-GGAGTAGAGTCAGTCACACCATTACA-3’) were used for DIG-labeled probes. Those probes were prepared according to manufacturer’s instruction (Roche, Tokyo, Japan). Whole mount *in situ* hybridization was performed according to Sive et al[8].

### Analysis of sperm motility

The P1-P3 peptides with partial amino acid sequences of SMIS were synthesized using a commercial service (Eurofins Genomics, Tokyo, Japan). An aliquot of sperm (1 μl) was suspended in modified Steinberg’s salt solution (ST: 58.2 mM NaCl, 0.67 mM KCl, 6 mM CaNO_3_, 0.83 mM MgSO_4_, 10 mM HEPES-NaOH; pH 8.5) or 10-fold diluted ST (1/10 ST) with double distilled water. Each synthetic peptide was prepared in one of the solutions at 1 μM to 10 mM. Sperm were observed at room temperature under a phase-contrast microscope (BX51, Olympus Co., Tokyo, Japan) equipped with a 10x or 20x objective, and recordings were made on a personal computer with a digital high-speed camera (HAS-220; DITECT, Tokyo, Japan). Motility was estimated based on the percentage of sperm showing undulation of the undulating membrane or progressive motility in *C*. *pyrrhogaster* at 1 min and 5 min; or stable and unstable rotary motion in *R*. *arboreus* at 5 min; or flagellar beating at a high and medium amplitude in *B*. *japonicus* at 1 min; or flagellar beating and progressive motility in *X*. *laevis* at 1 min. The significance of the differences was evaluated with Student’s t-test.

### Fluorescence staining

The fluorescein isothiocyanate-labeled P1 (FITC-P1) and FITC-P3 were synthesized by a commercial service (Eurofins Genomics Inc., Tokyo, Japan) and were incubated with sperm at 1 or 10 μg/ml for 5 min. To examine the immunolocalization of the SMIS in the oviduct-secreted matrix, the matrix obtained from oviductal lumen were fixed in methanol. For generation of a polyclonal antibody, referred to as the anti-P3 antibody, a synthetic peptide (YSLLKVTRSCS) including the P3 site was conjugated with keyhole limpet hemocyanin (KLH) and a rabbit was immunized with it. Immunogloblins were purified by the affinity to the synthetic peptide without KLH-conjugation. For immunostaining, the anti-P3 antibody (0.7 μg/ml) or the anti-SMIS antibody (1μg/ml) were used as primary antibodies and an AlexaFluor594-conjugated anti-rabbit IgG antibody (1 μg/ml) (Thermo Fisher Scientific, Waltham, MA) or an AlexaFluor488-conjugated anti-mouse IgG antibody (1 μg/ml) (Thermo Fisher Scientific, Waltham, MA) were done as secondary antibodies, respectively. In a control, FITC-P3 and the anti-P3 antibody were preabsorbed by the anti-P3 antibody and the peptide used to generate the anti-P3 antibody, respectively. The sperm and sections were observed with a confocal laser scanning microscope (c2; Nikon Co., Tokyo, Japan).

## Results

### Identification of the *SMIS* gene

**Fig 1:**
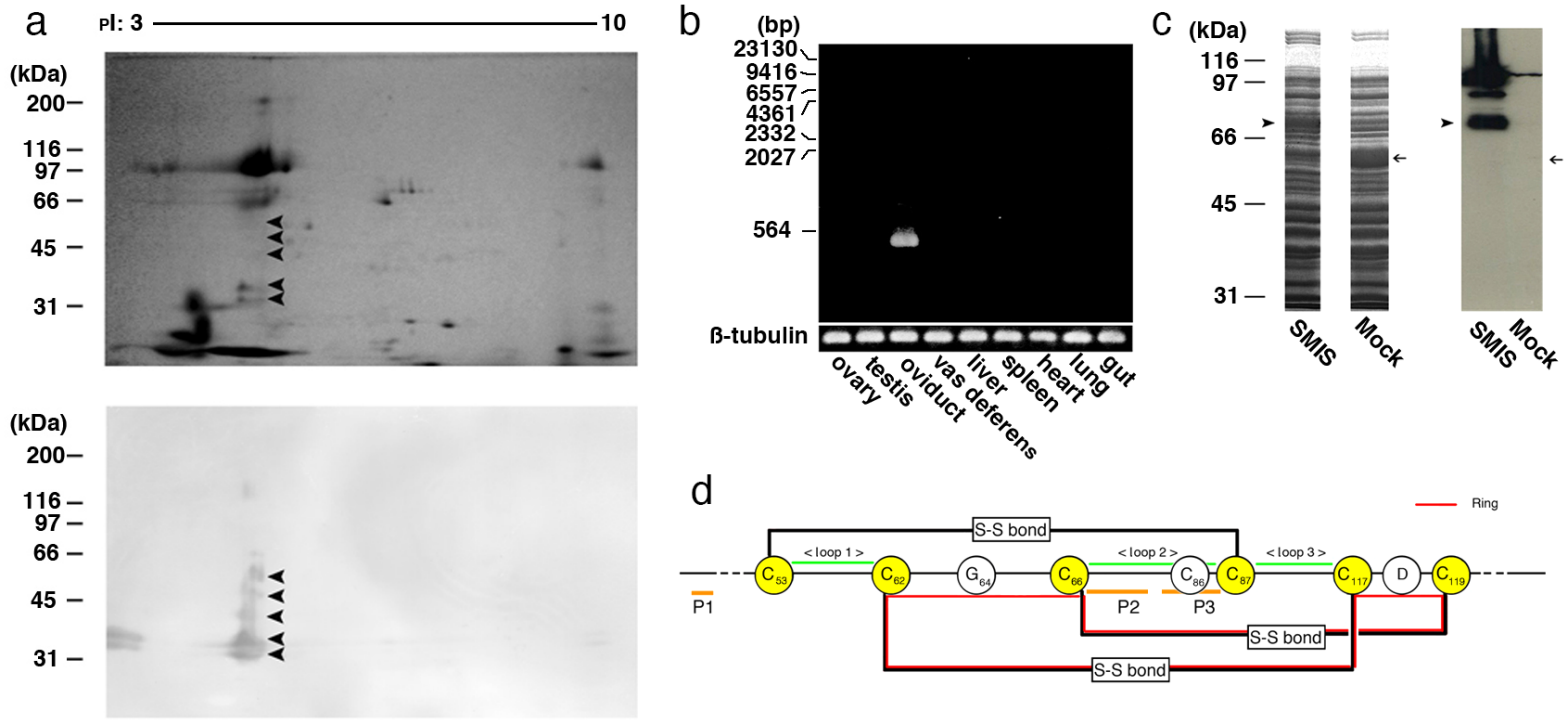
Identification and characterization of SMIS. (a) Immunoblotting of the SMIS protein in the JE of *C*. *pyrrhogaster*. Substances present in the JE were separated through 2D-electrophoresis and transferred to a PVDF membrane. Immunoreaction was performed using an anti-SMIS monoclonal antibody (mAb)[4]. The upper and lower photographs show Coomassie brilliant blue staining of the gel and an immunoblot image, respectively. Arrowheads indicate the SMIS proteins. (b) Amplification of *SMIS* cDNA through reverse transcription-polymerase chain reaction (RT-PCR). Total RNA was purified from the indicated organs using the TRIzol^®^ reagent (Invitrogen), and 1 μg of each sample was reverse-transcribed with an oligo-(dT) primer. Subsequently, PCR was performed using a primer set specific for the *SMIS* gene. In the control, primers specific for the *ß-tubulin* gene were used for PCR. (c) Immunoblotting of recombinant SMIS protein with an anti-SMIS mAb. Recombinant SMIS protein was produced in *Escherichia coli* by transfection of an expression vector (pCold TF, TaKaRa Bio, Tokyo, Japan) containing the open reading frame of *SMIS* cDNA. The protein was subjected to 1D-electrophoresis and immunoblotted with the anti-SMIS mAb. The left two columns show Coomassie Brilliant Blue staining of the acrylamide gel. The right shows specific binding of the anti-SMIS mAb. Arrowheads and arrows indicate the proteins produced from expression vectors with (SMIS) and without (Mock) *SMIS* cDNA, respectively. (d) A CK motif in the deduced amino acid sequence of the SMIS. The CK motif includes 6 cysteine residues at the 53rd, 62nd, 66th, 87th, 117th, 119th from N-terminus. Disulfide bonds are formed between cysteines-53 and -87, -62 and -117, and -87 and -119 to produce one ring and 3 loops (loops 1–3) that are supposed to be exposed on the outside. P1-P3 indicate the corresponding sites for preparation of the peptides for sperm motility analysis.

**Fig 2:**
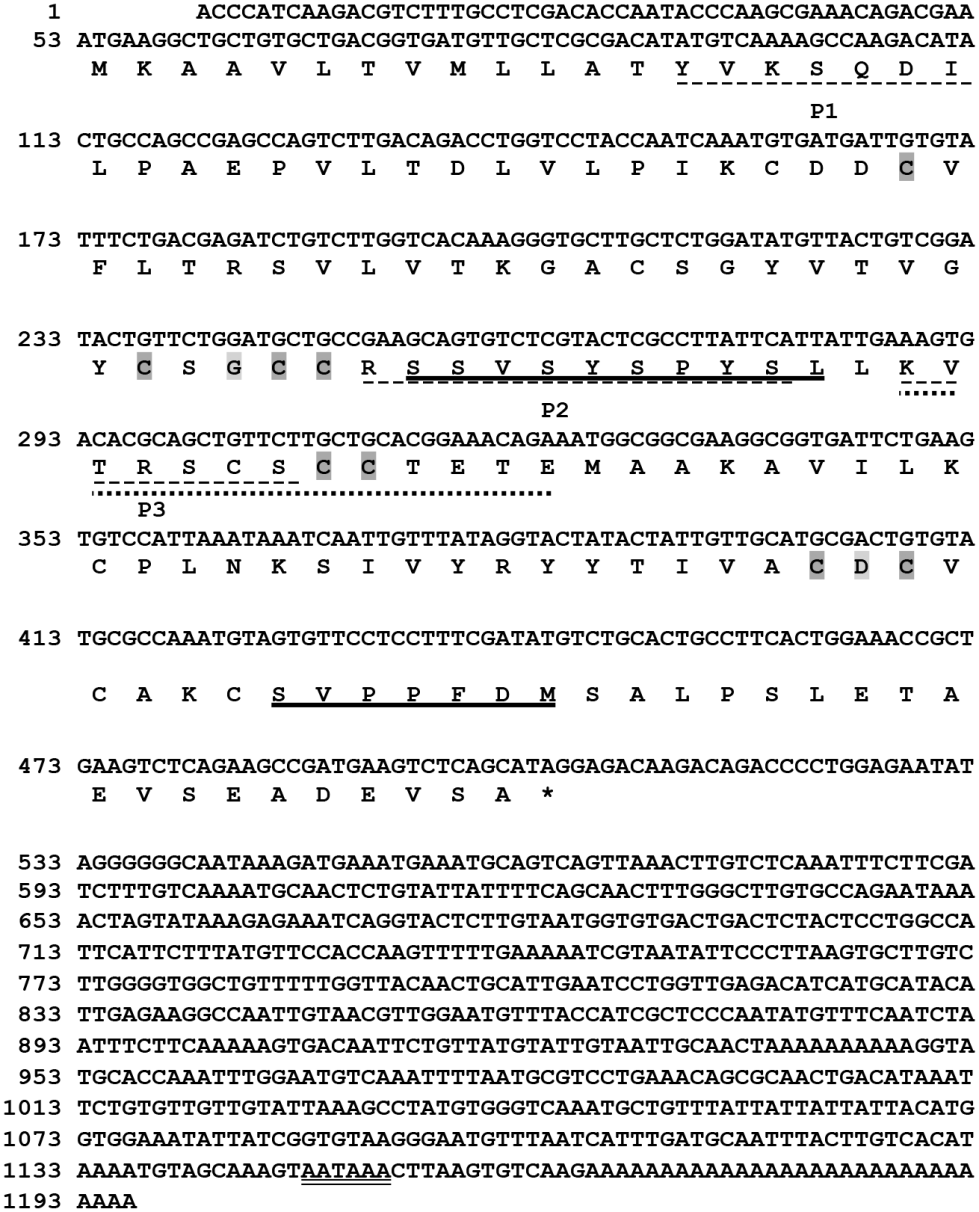
Base sequence of *SMIS* gene and its deduced amino acid sequence. Bold line indicates the amino acid sequences obtained by Edman degradation of the SMIS. Gray shade indicated the conserved amino acids in the CK motif of the matrix type[9]. Dotted line shows the sites of the P1-P3 peptides. The P3 peptides with different sizes have same effect on motility. Doubled underline shows polyadenylation signal.

**Fig 3:**
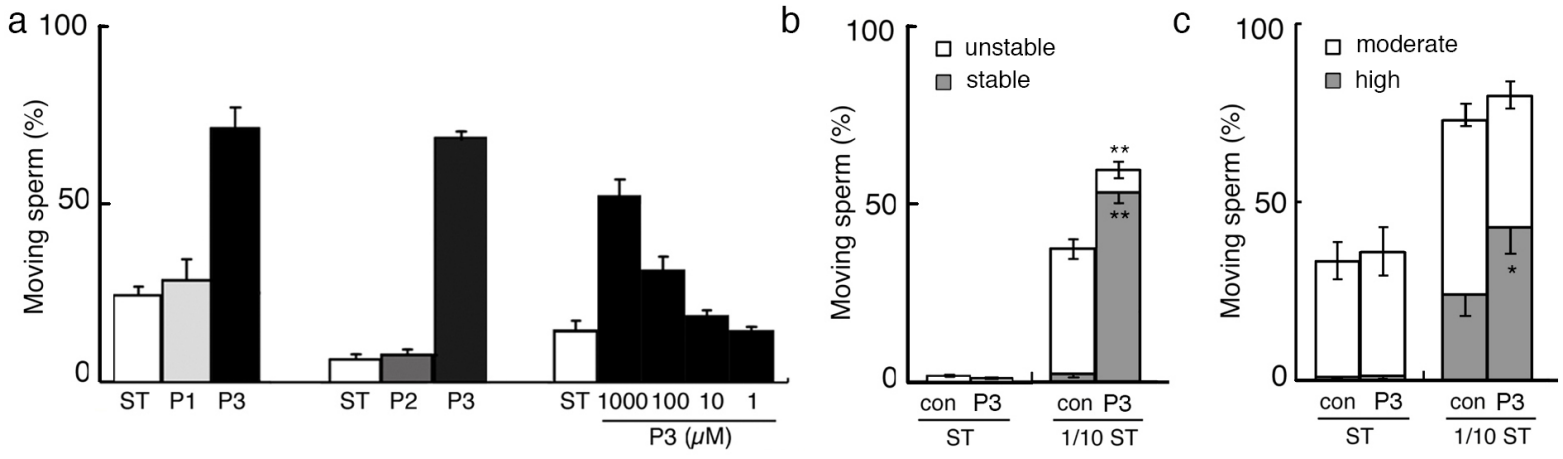
Motility initiation of *C*. *pyrrhogaster* sperm by the P3 peptide in isotonic solution. (a) Sperm were suspended in modified Steinberg’s salt solution (ST) containing 1 mM P1, P2, or P3. The percentages of sperm showing circular motion were estimated over 5 min. Dose-dependent P3-initiated sperm motility was induced within the range of 1μM-1mM. (b and c) Sperm of *R*. *arboreus* (b) or *B*. *japonicus* (c) were suspended in ST or 1/10 ST (1 mM for *R*. *arboreus* or 10 mM for *B*. *japonicus*). (b) Percentages of motile sperm showing stable or unstable rotary motion over 5 min. (c) Percentages of motile sperm whose flagellum beat with a high amplitude or moderate amplitude over 1 min. Asterisks and double asterisks indicate significant differences at p<0.05 and p<0.01, respectively, against 1/10 ST by Student’s t-test.

**Fig 4:**
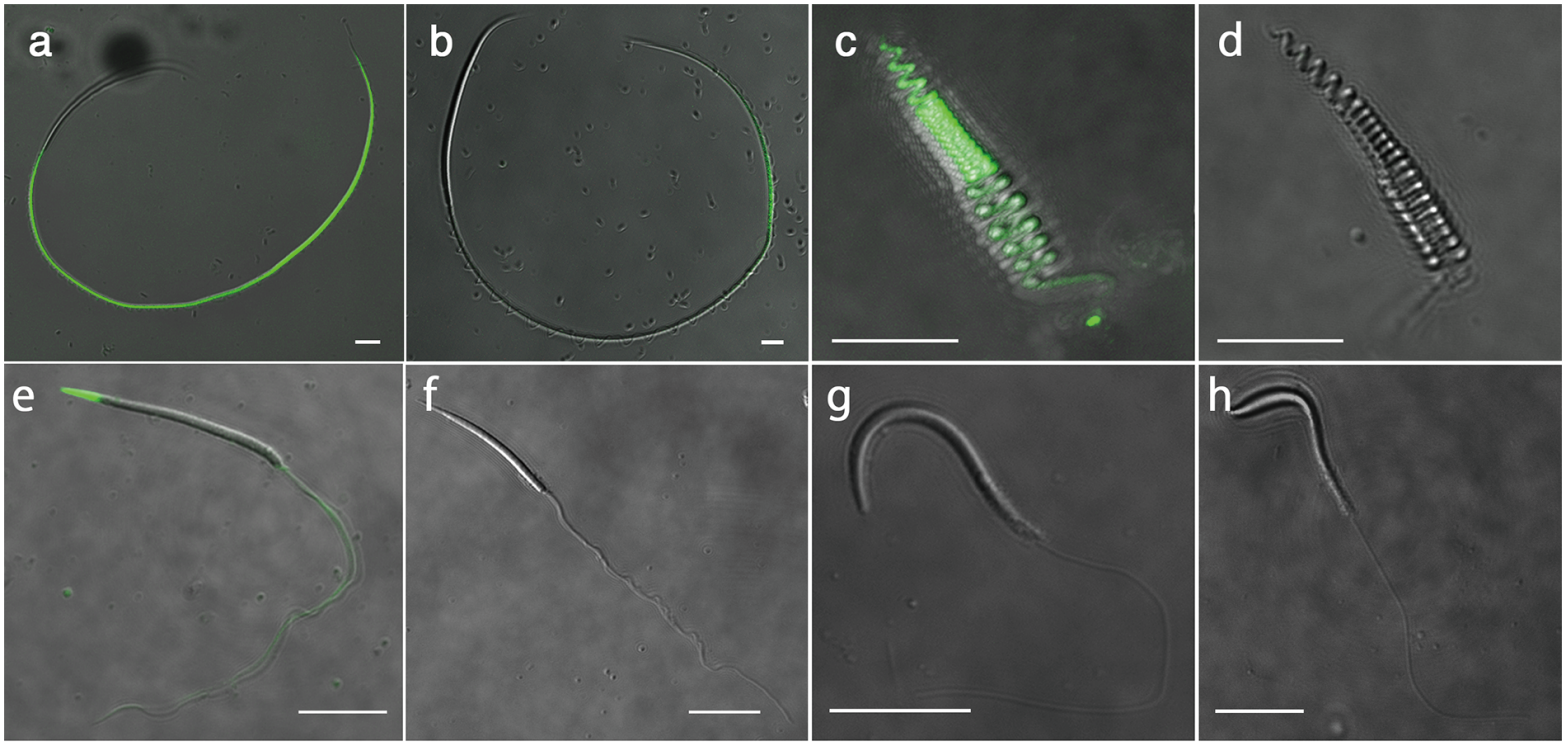
P3 peptide binding to sperm. Sperm of *C*. *pyrrhogaster* (a, b), *R*. *arboreus* (c, d), *B*. *japonicus* (e, f), and *X*. *laevis* (g, h) were treated with FITC-P3 (1 mM in a, c, g and 10mM in **e**) or FITC-P1 (1 mM in b, d, h and 10 mM in f). Bar: 10 μm.

Immunoblotting of an egg jelly extract with the anti-SMIS antibody[4] revealed several spots at isoelectric point 4.3 (Fig 1a), and partial amino acid sequences of PVPYPSYPL and PVSSFDM were obtained from the smallest spot via Edman degradation. Through reverse transcription-polymerase chain reaction using degenerate primers corresponding to those sequences, a 1196-base pair cDNA sequence encoding a 150-amino acid sequence was finally obtained from the total RNA of oviductal cells (Fig 2; accession number: LC026154). The candidate *SMIS* gene was specifically expressed in epithelial secretory cells in oviduct (Fig 1b and S1 Fig), and the anti-SMIS antibody specifically recognized the recombinant protein (Fig 1c). No genes with substantial similarity were identified in the NIH gene and protein data bank and the Xenbase, which include full genome data of all amphibian model animals, although the deduced sequence showed relatively high similarity to a C-terminal region of mucin 5 (S1 Table). It contained 14 cysteine residues, 6 of which could form a cysteine-knot (CK) motif (Figs 1d and 2). The CK motif is present in many secreted proteins, such as mucins and Wnt inhibitors, and it mediates polymerization to stabilize their three-dimensional conformation[9,10]. Agglutinated conformation of the SMIS seen in Fig 1a and 1c is thought to attribute to this motif. In the Wnt inhibitors Sclerostin and Wise, the 2nd loop of the CK motif binds to the receptor, resulting in their inhibitory activity[10]. Thus, we synthesized 3 oligo-peptides with the deduced amino acid sequences of the N-terminal and C-terminal regions of the 2nd loop (designated P2 and P3, respectively) and the N-terminal region of SMIS (designated P1) (Fig 2). Among these peptides, only P3 induced waving of the undulating membrane, in which an axoneme is present to provide thrust at the sperm tail (Fig 3a and S1 Movie). These sperm immediately acquired progressive motility with a circular trajectory, which is typically induced by SMIS in egg jelly extracts[4] (S2 Movie). This result indicates that C-terminal region of the CK motif is an active site of the candidate protein that can be completely substituted for SMIS. FITC-P3 bound to the axial rod of the tail in *C*. *pyrrhogaster* sperm (Fig 4a), whereas FITC-P1, which was used as a control, bound to the axial rod of the posterior region of the tail, termed the principal piece (Fig 4b). The binding of the FITC-P3 to the axial rod of the tail was also blocked by the anti-P3 antibody except for that of the principal piece (S2 Fig), thus indicating that the P3 specifically binds to the axial rod of the anterior region of the tail, termed the midpiece. Midpiece is significant for the regulation of sperm motility induced by the SMIS[11]. These results indicate that the obtained cDNA encodes SMIS, whose active site is located at the C-terminal region of the 2nd loop of the CK motif (Figs 1d and 2).

### Conserved effect of SMIS in amphibians

**Fig 5:**
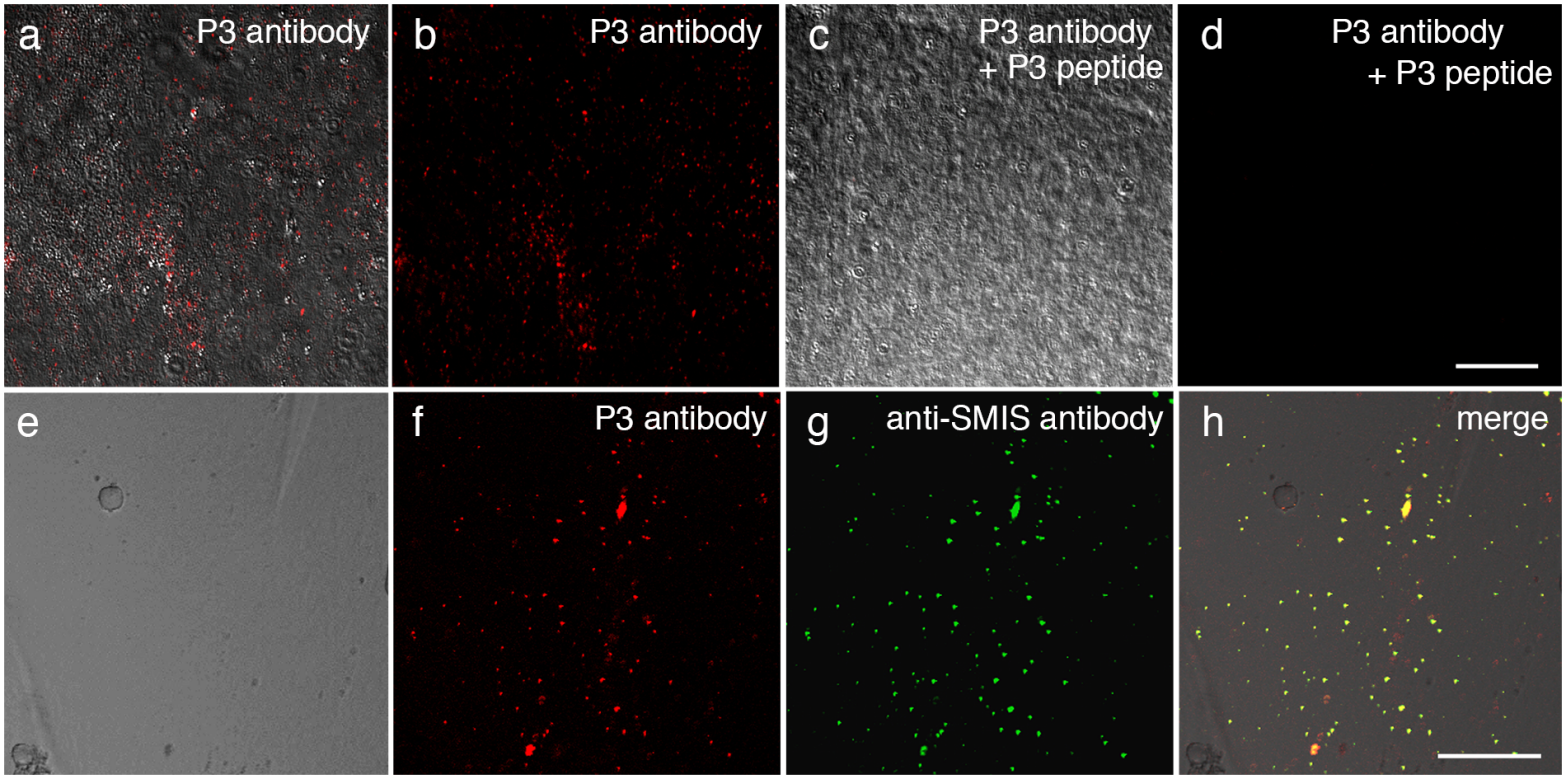
Immunolocalization of SMIS in the oviduct-secreted matrix of *R*. *arboreus*. Egg coat matrix of *R*. *arboreus* was fixed in methanol and immunostained with the anti-P3 antibody or the anti-SMIS antibody. (a-d) Fluorescence staining with the anti-P3 antibody (a-c) and the anti-P3 antibody preabsorbed by the synthetic peptide including P3 site as a control (d). (e-h) Double-staining with the anti-P3 antibody (f) and the anti-SMIS antibody (g). (h) Merged image. Bar: 200 μm (d) and 50 μm (h).

To address the function of SMIS in external fertilization, the effect of P3 on sperm motility was examined in two anuran species with different reproductive modes. *R*. *arboreus* forms a foam nest on a tree in which eggs are deposited and fertilized with sperm. The foam nest is composed of a highly viscous matrix derived from an oviductal secretion. The sperm of this species exhibit a rotary motion that is specifically adapted to the foam nest matrix[12]. *R*. *arboreus* sperm were observed to initiate motility in a hypotonic solution, but the rotary motion was not steadily maintained (Fig 3b). P3 significantly increased the motility rate and enhanced motility to maintain continuous rotation of motile sperm (S3 Movie), although it was not effective in an isotonic solution. FITC-P3 bound to all areas of *R*. *arboreus* sperm (Fig 4c) in contrast that no binding of FITC-P1 or FITC-P3 preabsorbed by the anti-P3 antibody was observed (Fig 4d and S2 Fig). The anti-P3 antibody was specifically bound to the oviduct-secreted matrix that forms the foam nest of *R*. *arboreus* (Fig 5). These results indicate that in *R*. *arboreus*, SMIS enhances the motility of motile sperm and the initiation of sperm motility, but with a strict dependency on a hypotonic environment.

Enhancement of motility was similarly observed in the sperm of *B*. *japonicus*. Although *B*. *japonicus* sperm did not exhibit forward motility in the isotonic solution, the motility rate was increased by hypoosmolality, and P3 significantly strengthened high-amplitude flagellar beating to enhance the vibratory motion of the motile sperm (Fig 3c and S4 Movie). High-amplitude flagellar beat is beneficial for propulsion in a viscous matrix[13], suggesting that the strengthened flagellar beating of *B*. *japonicus* sperm occurs to allow penetration of a jelly matrix. FITC-P3 bound to the tip of the head and axial fiber of the tail in *B*. *japonicus* sperm (Fig 4e). Preabsorption of FITC-P3 by the anti-P3 antibody weakened but not completely blocked the binding probably because of technical difficulty in association with the requirement of relatively high FITC-P3 concentration (10 μg/ml). However, no binding of the anti-P3 antibody was detected in the egg coat matrix of *B*. *japonicus*. We speculate that the egg coat of *B*. *japonicus* exhibits a very small amount or a specific conformation of SMIS that was undetectable through immunocytochemistry with the anti-P3 antibody.

These results suggest that in the two externally-fertilizing anurans, SMIS enhances the motility of motile sperm, rather than initiating sperm motility, to allow the sperm to penetrate the oviduct-derived egg coat matrix. Enhancement of motility by SMIS was also observed in *C*. *pyrrhogaster* sperm in a hypoosmotic environment. Most sperm initiated undulation of their undulating membrane in a hypotonic solution, but many of them did not show progressive motility (S3 Fig). P3 enhanced the undulation of the undulating membrane in a hypotonic solution, leading to strong progressive motility with a circular trajectory in most sperm, like P3-treated sperm in an isotonic solution. This finding suggests that in the natural fertilization of *C*. *pyrrhogaster*, SMIS simultaneously initiates and enhances sperm motility on the surface of the egg jelly, which allows sperm to penetrate the jelly matrix. Thus, the common effect of SMIS in anuran and urodele amphibians is to enhance sperm motility to allow penetration of the egg coat.

### SMIS is ineffective in *X*. *laevis* sperm

*X*. *laevis* is the amphibian model organism, and the full genomic data of this species was recently established. In a survey of the *X*. *laevis* genome database (J-strain 8.0 genome), no sequences homologous to SMIS were identified, based on an e-value of less than 0.01 using the tblastn protocol with the BLOSUM62 matrix. Thus, it was confirmed that the *SMIS* gene has been lost in the genome of *X*. *laevis*. *X*. *laevis* sperm initiated motility in a hypotonic solution (S4 Fig), similar to those of *R*. *arboreus* and *B*. *japonicus*. However, P3 did not increase the proportion of motile sperm or that of sperm with strengthened motility in either the hypotonic or isotonic solution (S4 Fig). The ineffectiveness of P3 was supported by the observation that neither FITC-P3 nor -P1 bound to *X*. *laevis* sperm (Fig 4g and 4h). These results indicate that SMIS does not act in the fertilization of *X*. *laevis*.

## Discussion

Sperm motility is regulated by various factors such as osmotic changes[14], cations[15], steroids[16,17] and peptides/proteins[4,18–23], each of which acts in a highly adaptive manner to species-specific fertilization environment. In the internal fertilization, an endopeptidase and progesterone are reported to initiate and hyperactivate sperm motility in the female reproductive tracts of silkworm and human, respectively[17,20]. SMIS is a new signal protein that initiates motility of the sperm stored by females in the internal fertilization.

Cysteine knot motif is widely conserved in extracellular molecules such as TGF-ßs, glycoprotein hormones, Wnt inhibitors, and mucins[9]. Six conserved cysteine residues in the CK motif form 3 disulfide bonds to cause 2 rings and 3 loops, and high hydrophobic regions in the loops contribute to dimerization/polymerization of the CK proteins. The CK motif of the SMIS showed high similarity to mucin 5 (S1 Table), suggesting the origin of this protein from the extracellular matrix. The SMIS includes hydrophobic regions in the 1st and 3rd loops of the CK motif (S5 Fig) and possesses an additional cysteine residue adjacently to the 4th conserved cysteine residue (Figs 1d and 2). That cysteine residue is common in the CK motif of mucins and forms an intermolecular covalent bond to stabilize dimer/polymer conformation[24,25]. These molecular features are thought to be responsible for the association of SMIS to form extracellular granules that are critical for the initiation of sperm motility on the surface of egg jelly matrix in the internal fertilization of *C*. *pyrrhogaster*[4]. On the other hand, an active site of the SMIS is adjacently to the additional cysteine residue in the C-terminal region of the 2nd loop (Fig 1d). The SMIS is suggested to initiate sperm motility through an activation of the SMIS itself[26], which is unique to the SMIS signal. It is supposed that the association of the SMIS sequesters the active site to make it inactive until the SMIS is activated.

The results of the present study indicate that SMIS in the egg coat matrix enhances sperm motility to allow penetration of the egg coat during fertilization in two evolutionarily advanced species of anurans, *R*. *arboreus* and *B*. *japonicus*, and the urodele *C*. *pyrrhogaster* (Fig 3a–3c and S3 Fig). Because *SMIS* gene was not identified in the gene data of any model organisms, but active SMIS is present in the egg coat matrix of primitive anuran and urodele species[6,7], SMIS is suggested to be a ligand for penetration of the egg coat that was evolved in anurans and urodeles. It is known that allurin, a sperm chemoattractant in the egg coat of *X*. *laevis*[23] is also an amphibian-specific protein. The amphibian egg coat, which is composed of thickly accumulated oviductal matrix, plays critical roles in fertilization and embryonic development, such as acting as a source of ligands and Ca^2+^ for the sperm-egg interaction[23,27], allowing attachment to substrates to sustain fertilized eggs[27] and providing protection against bacterial invasion of the developing embryo[27]. Furthermore, the egg coat shows a variety of morphologies and physicochemical properties depending on the species, which contribute to the success of reproduction in specific outside environments. However, the thick and viscous matrix of the egg coat prevents sperm with weak motility from propelling through it to access the egg. It is therefore reasonable that a signal for the enhancement of sperm motility, SMIS evolved based on the extracellular matrix protein such as mucin 5 (S1 Table) and has been widely conserved in amphibian fertilization.

SMIS signal shows apparent differences in the sites of stimulation by SMIS (Fig 4) and in the localizations of the SMIS in egg coat matrix[4] (Fig 5). The differences in the stimulation site are thought to be correlated with the development of forward motility in a species-specific egg coat matrix. The localization of the SMIS on the surface of egg jelly of *C*. *pyrrhogster*[4] is efficient to initiate motility of sperm quiescently stored by females, whereas uniform distribution of the SMIS in the matrix of *R*. *arboreus* (Fig 5) is reasonable because females mix spawned sperm into the egg coat matrix in the onset of fertilization. The responsiveness of sperm to SMIS also varies in association with the osmotic environment (Fig 3). In primitive amphibians, SMIS is effective in an isotonic environment[6,7]. This feature is conserved in advanced species of urodeles and enables SMIS to play the additional role of initiating sperm motility without hypoosmolality[3]. Conversely, the sperm of advanced anurans shows no response to SMIS in an isotonic solution (Fig 3). The effect of SMIS in an isotonic environment is not necessary for external fertilization, which might have resulted in the loss of this ability. Furthermore, SMIS have been lost in the fertilization of *X*. *laevis* (Fig 4g and S4 Fig). The morphological and physicochemical nature of the egg coat might be modified in this species to allow sperm penetration without motility enhancement by SMIS. Alternatively, loss of sperm responsiveness to hypoosmolality is reported in some terrestrial anurans[28]. Conclusively, diversification of reproductive mode often accompanied the restrictive modification of the mechanism for initiation and enhancement of sperm motility in amphibians. As the result of such modification of the mechanism for sperm motility initiation/enhancement, SMIS might have become unnecessary in the internal fertilization of higher vertebrates.

SMIS enhanced the unique motions of the sperm of *C*. *pyrrhogaster* (S1 Movie) and *R*. *arboreus* (S3 Movie), which are based on their specific morphologies, of *C*. *pyrrhogaster* sperm being characterized by a stiff axial rod attaching the undulating membrane to one side of the tail[29], and *R*. *arboreus* sperm done by a screw-shape with doubled axonemes in the tail[30]. As the sperm of both species can move forward only in a viscous matrix[12,29], their unique sperm morphologies are related to the penetration of the egg coat matrix that is optimized for fertilization and embryonic development either inside the female's body or on a tree. These characteristics suggest the possible relationship between the egg coat matrix and sperm morphology during the diversification of reproductive modes: the nature of the matrix is modified adaptively to a specific reproductive mode, whereas sperm morphology is selectively altered to allow forward motility in the modified matrix. This possibility is supported by the fact that sperm morphology is highly and non-phylogenetically varied in anurans[31], whose reproductive modes have diversified with adaptation to various outside environments[1], in contrast to the lower variation observed in urodeles[32], in which the mode of reproduction is largely restricted to internal fertilization[27,33]. The evolution of SMIS signal might facilitate the adaptive modification of egg coat and the selective alteration of sperm morphology to finally establish internal fertilization in urodele amphibians.

## Supporting Information

S1 Fig *SMIS* gene expression in oviductal cells detected by *in situ* hybridization. (a) Antisense probe. Signals were in cytoplasm filled with many secretory vesicles. (b) Sense probe. Dashed lines indicate the borders of individual epithelial secretory cell. Asterisks indicate the lumen of oviduct. n: nuclei. Bar: 20 μm.
(TIF)

S2 Fig Biding of FITC-P3 to sperm. *C*. *pyrrhogaster* sperm (a, b) and *R*. *arboreus* sperm (c, d) were stained with FITC-P3 (a, c) or FITC-P3 preabsorbed by the anti-P3 antibody (b, d). Bar: 50 μm (a, b) and 10 μm (c, d).
(TIF)

S3 Fig Motility initiation of *C*. *pyrrhogaster* sperm by the P3 peptide in hypotonic solution. Sperm were suspended in 1/10 ST containing 1 mM P3. JE and ST were used as positive and negative controls, respectively. The percentages of sperm with circular motion were examined over 1 and 5 min. No sperm with a circular motion were observed in ST, and sperm with a circular motion were induced in JE in 5 min, which is in accord with previously reported results[4]. Note that sperm with a circular motion were induced by P3 in hypotonic 1/10 ST. Asterisks indicate significant differences (p<0.01) against ST by Student’s t-test.
(TIF)

S4 Fig Effects of P3 on the sperm motility of *X*. *laevis*. *X*. *laevis* sperm were suspended in ST or 1/10 ST containing 1 mM P3. (a) The relative percentage of sperm with a beating flagellum versus those in a test solution, which was designated as the motility index. (b) Percentages of sperm with progressive motility versus sperm with a beating flagellum.
(TIF)

S5 Fig Kyte-Doolittle hydrophobicity plot of the SMIS. (TIF)

S1 Movie Motility-initiated sperm of *C*. *pyrrhogaster* in modified Steinberg’s salt solution containing 1 mM P3 peptide. (MOV)

S2 Movie Motility-initiated sperm of *C*. *pyrrhogaster* in JE. (MOV)

S3 Movie Motility-enhanced sperm of *R*. *arboreus* in 10-fold-diluted modified Steinberg’s salt solution containing 1 mM P3 peptide. (MOV)

S4 Movie Motility-enhanced sperm of *B*. *japonicus* in 10-fold-diluted modified Steinberg’s salt solution containing 10 mM P3 peptide. (MOV)

S1 Table Proteins showing similarity to SMIS with highest e-value by the Basic Local Alignment Search Tool. (TIF)
