# Supplementary figures and images for: A Novel Cysteine Knot Protein for Enhancing Sperm Motility That Might Facilitate the Evolution of Internal Fertilization in Amphibians
Source: PLoS One. 2016 Aug 31;11(8):e0160445. doi: 10.1371/journal.pone.0160445 (PMC5007030; doi:10.1371/journal.pone.0160445)

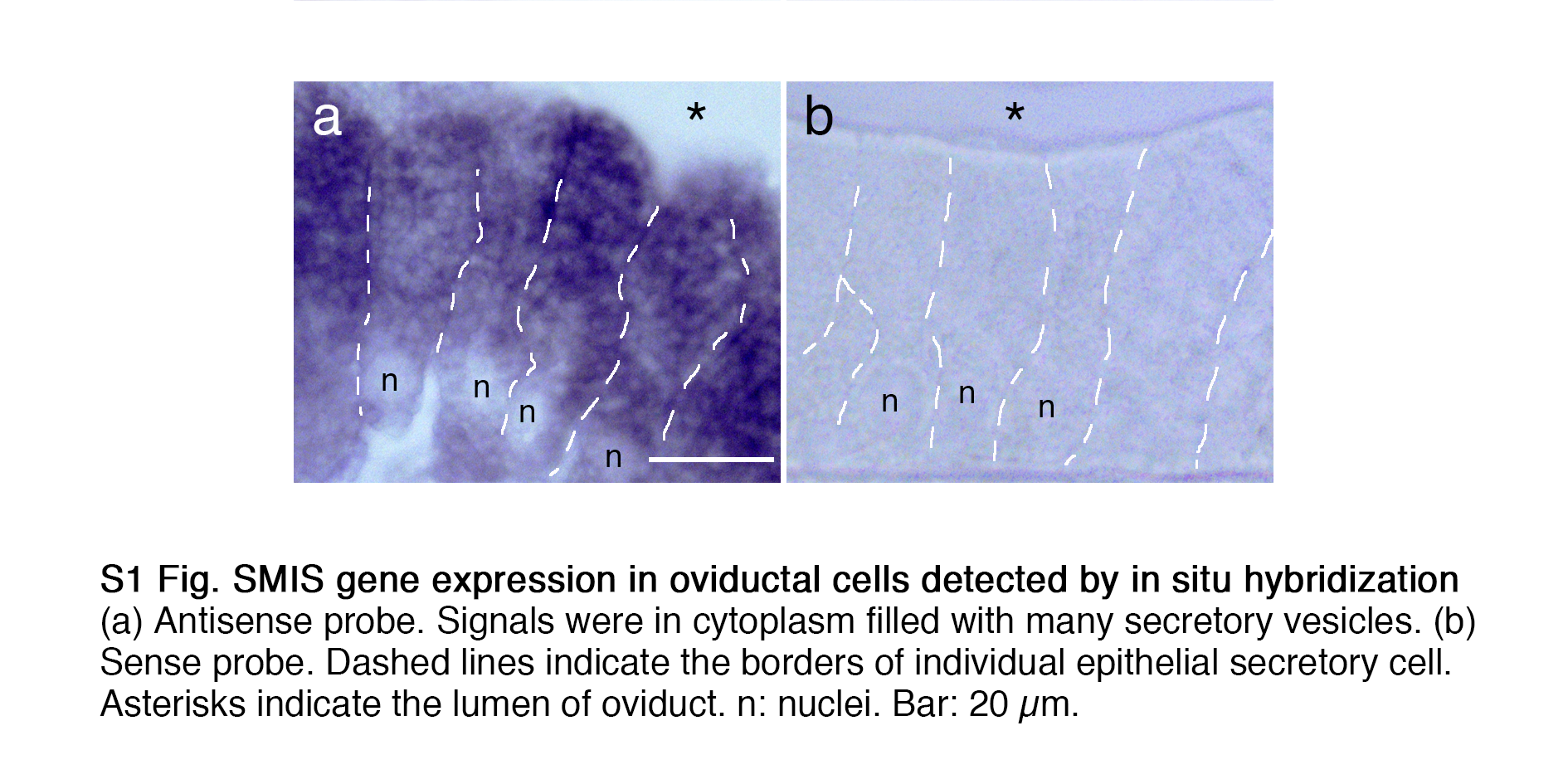

Supplement: S1 Fig — (a) Antisense probe. Signals were in cytoplasm filled with many secretory vesicles. (b) Sense probe. Dashed lines indicate the borders of individual epithelial secretory cell. Asterisks indicate the lumen of oviduct. n: nuclei. Bar: 20 μm. (TIF) [file pone.0160445.s001.tif]

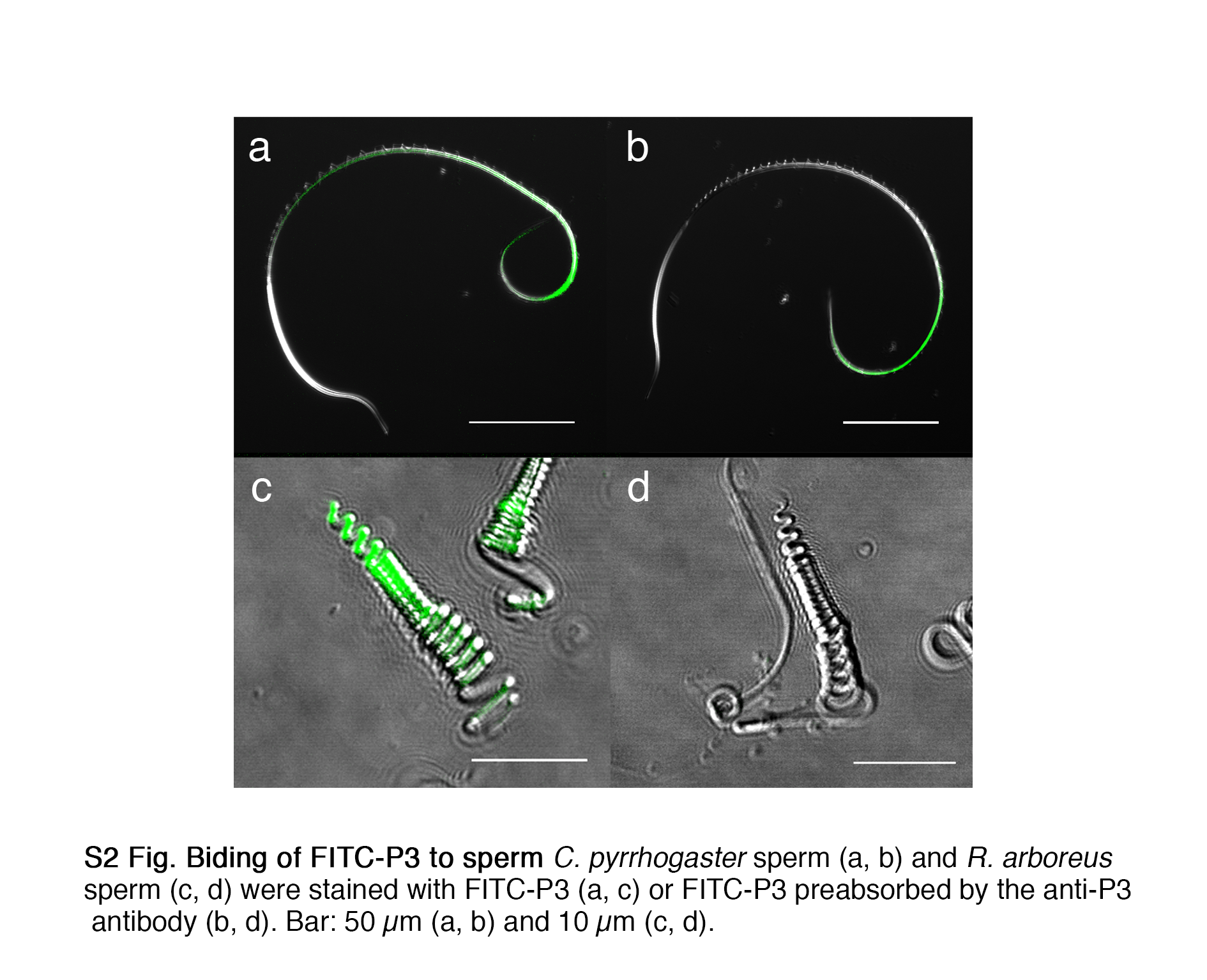

Supplement: S2 Fig — C. pyrrhogaster sperm (a, b) and R. arboreus sperm (c, d) were stained with FITC-P3 (a, c) or FITC-P3 preabsorbed by the anti-P3 antibody (b, d). Bar: 50 μm (a, b) and 10 μm (c, d). (TIF) [file pone.0160445.s002.tif]

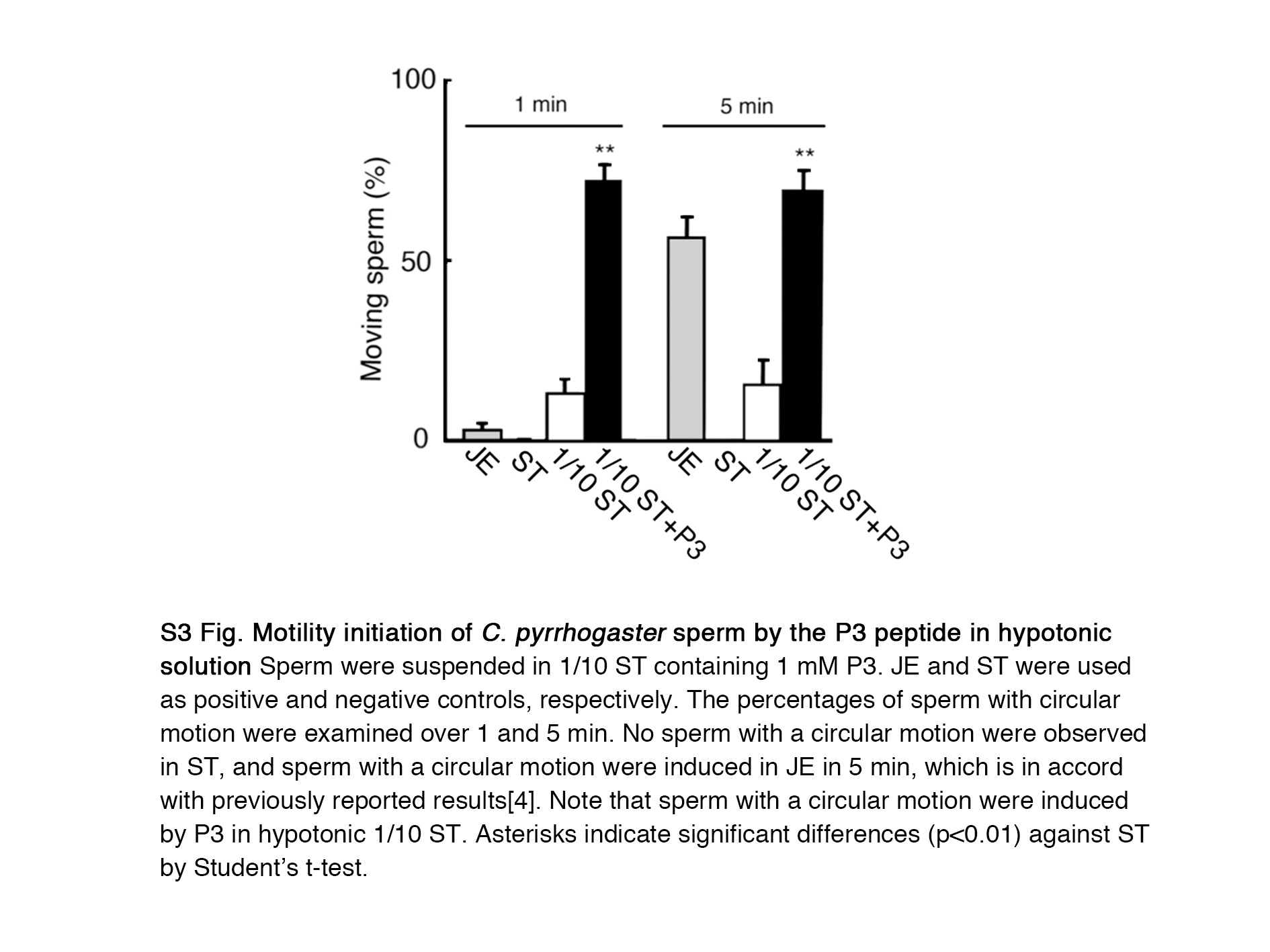

Supplement: S3 Fig — Sperm were suspended in 1/10 ST containing 1 mM P3. JE and ST were used as positive and negative controls, respectively. The percentages of sperm with circular motion were examined over 1 and 5 min. No sperm with a circular motion were observed in ST, and sperm with a circular motion were induced in JE in 5 min, which is in accord with previously reported results[4]. Note that sperm with a circular motion were induced by P3 in hypotonic 1/10 ST. Asterisks indicate significant differences (p<0.01) against ST by Student’s t-test. (TIF) [file pone.0160445.s003.tif]

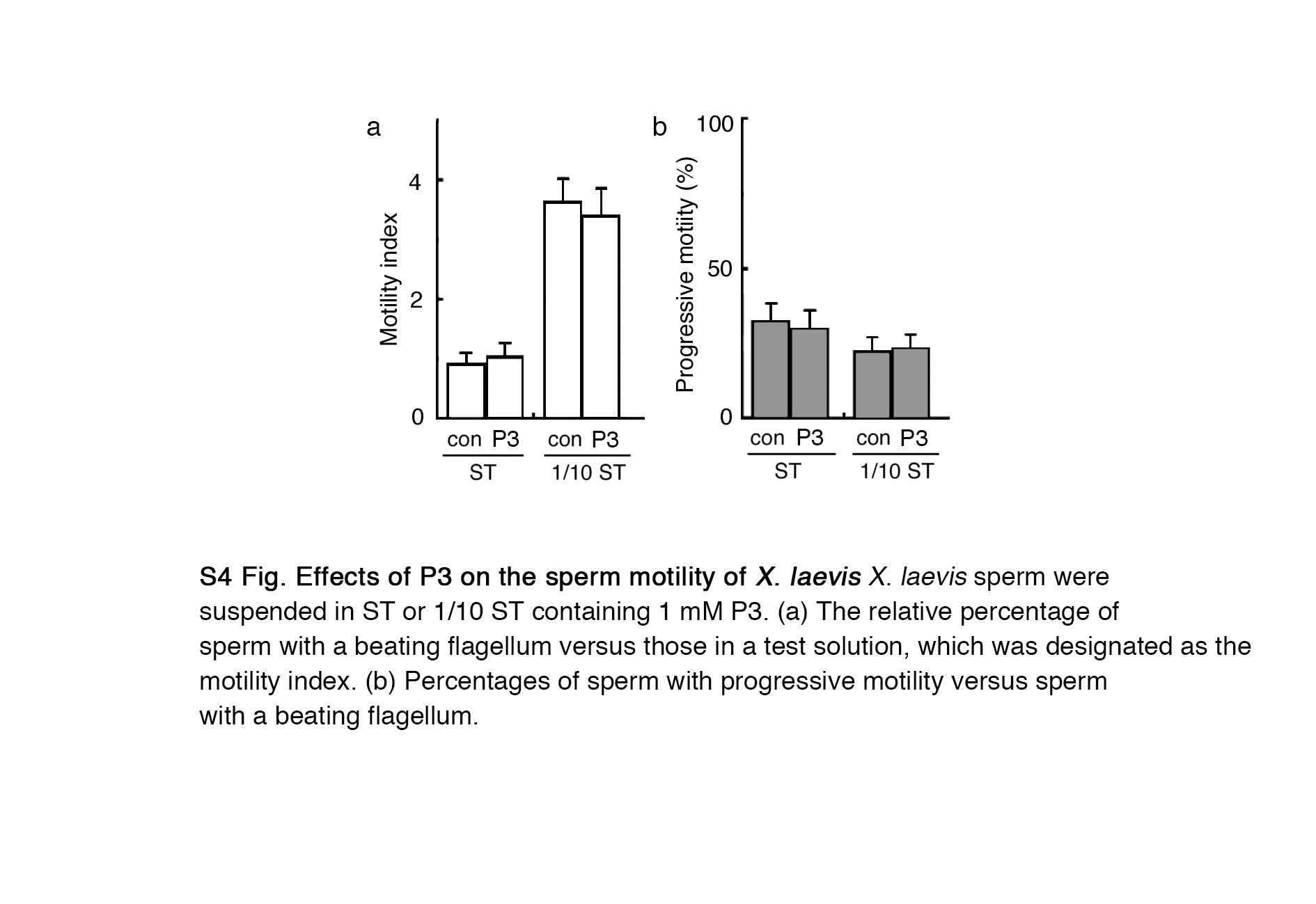

Supplement: S4 Fig — X. laevis sperm were suspended in ST or 1/10 ST containing 1 mM P3. (a) The relative percentage of sperm with a beating flagellum versus those in a test solution, which was designated as the motility index. (b) Percentages of sperm with progressive motility versus sperm with a beating flagellum. (TIF) [file pone.0160445.s004.tif]

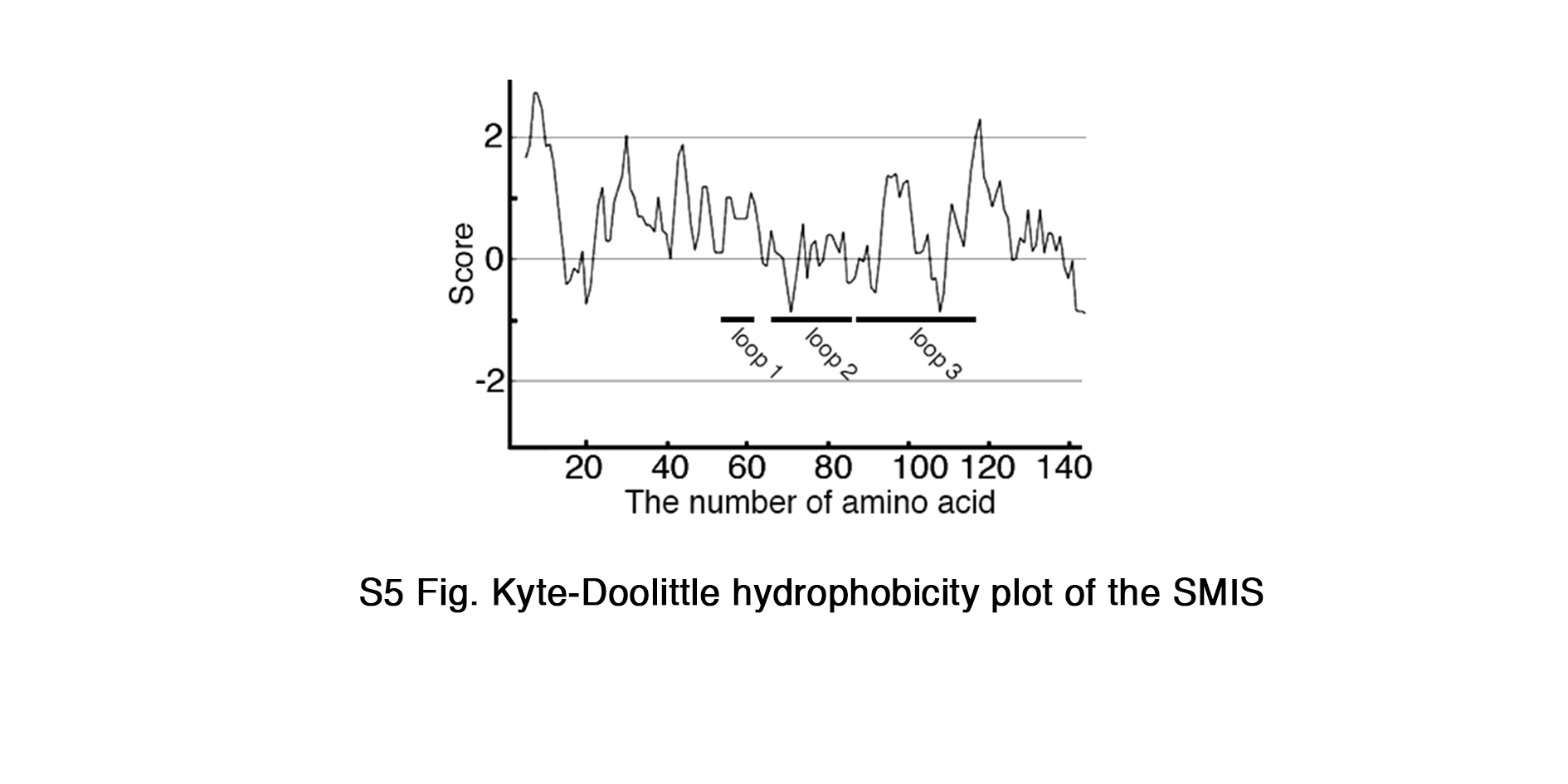

Supplement: S5 Fig — (TIF) [file pone.0160445.s005.tif]

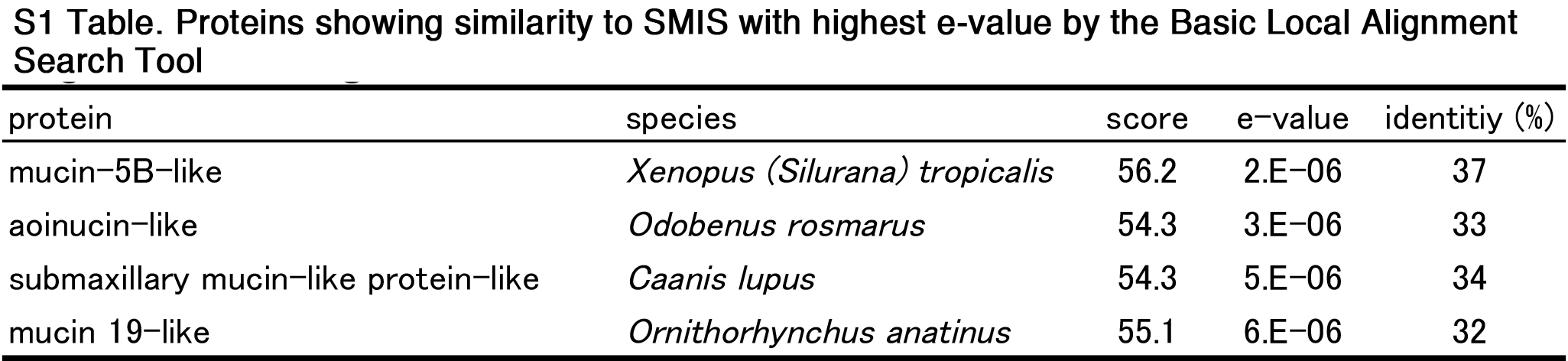

Supplement: S1 Table — (TIF) [file pone.0160445.s010.tif]
